# Effects of pollution, low temperature and influenza syndrome on the excess mortality risk in winter 2016–2017

**DOI:** 10.1186/s12889-019-7788-8

**Published:** 2019-11-04

**Authors:** Rossella Murtas, Antonio Giampiero Russo

**Affiliations:** Epidemiology Unit, Agency for Health Protection (ATS) of Milan, Corso Italia 19 –, 20122 Milan, Italy

**Keywords:** Air pollution, Low ambient temperature, Influenza, Natural mortality, Cardiovascular mortality, Respiratory mortality, Case-crossover

## Abstract

**Background:**

In the winter of 2016–2017, the number of deaths recorded in the north-west Europe was significantly higher than that in previous years. This spike in mortality was attributed principally to an influenza epidemic, but the contribution of air pollution and cold temperature has not been investigated. Information on the combined effect of low temperatures, influenza epidemic, and air pollution on mortality is inadequate. The objective of this study was to estimate the excess mortality in the winter of 2016–2017 in the metropolitan area of Milan, and to evaluate the independent short-term effect of 3 risk factors: low temperatures, the influenza epidemic, and air pollution.

**Methods:**

We used a case-crossover, time-stratified study design. Mortality data were collected on all people aged > 65 years who died of natural causes, due to respiratory diseases or cardiovascular diseases, between December 1, 2016 and February 15, 2017. Environmental data were extracted from the Regional Environmental Protection Agency. The National Surveillance Network provided data on influenza epidemic.

**Results:**

Among the 7590 natural deaths in people aged > 65 years, 965 (13%) were caused by respiratory conditions, and 2688 (35%) were caused by cardiovascular conditions. There were statistically significant associations between the minimum recorded temperature and deaths due to natural causes (OR = 0.966, 95% CI: 0.944–0.989), and cardiovascular conditions (OR = 0.961, 95% CI: 0.925–0.999). There were also statistically significant association between the influenza epidemic and deaths due to natural causes (OR = 1.198, 95% CI: 1.156–1.241), cardiovascular conditions (OR = 1.153, 95% CI: 1.088–1.223), and respiratory conditions (OR = 1.303, 95% CI: 1.166–1.456). High levels of PM10 (60 and 70 μg/m^3^) were associated with a statistically significant increase in natural and cause-specific mortality. There were statistically significant interactions between PM10 and influenza for cardiovascular-related mortality, and between influenza and temperature for deaths due to natural causes.

**Conclusions:**

Excess of mortality in Milan during winter 2016–2017 was associated with influenza epidemic and concomitant environmental exposures, specifically, the combined effect of air pollution and low temperatures. Policies mitigating the effects of environmental risk factors should be implemented to prevent future excess mortality.

## Background

In the last few decades, environmental actors, such as meteorological conditions and air pollution, have been shown to have an effect on mortality.

Air pollution is currently considered the most significant environmental cause of disease, with 4.2 million premature deaths related to air pollution in 2016 [1]. The differential effect of air pollution on health, which is particularly deleterious for older people and people with limited resources, is of major concern to global health organizations [2]. Large, multi-city studies in Europe, USA, and other parts of the world, have shown that ambient air pollution has adverse effects on total and cause-specific morbidity and mortality [3–5]. Outdoor fine particulate air pollution is estimated to be responsible for about 3% of global adult cardiopulmonary-related mortality [6]. Air pollution is likely to have similar adverse effects in developing countries, with Asian countries contributing approximately with two-thirds of the global burden [6]. In Italy, the EpiAir project [7], which is a project dedicated to measuring the impact of air pollution on health, has suggested that air pollution has an immediate effect on the number of natural deaths from all causes combined, and the number of deaths attributed to cardiovascular and respiratory conditions. In addition, the European ESCAPE study, estimated the effects of chronic air pollution using data from previous cohort studies. The authors’ findings confirmed the relationship between mortality and long-term exposure to particulate matter, fine particles, and nitrogen compounds; moreover, the authors also reported an association between these risk factors and invasive lung neoplasms [8].

However, air pollution is not the only environmental risk factor associated with premature deaths. Cold temperatures may also have an effect on mortality, especially from cardiovascular and respiratory causes [9–11]. A Portuguese study on the impact of cold temperature on the risk of death from natural, cardiovascular, and respiratory conditions, showed that the effect of cold temperatures on mortality had a 1–2-day lag period, and that risk peaked after 6–7 days after the onset of the cold weather, and remained elevated for up to 28 days [10]. Cold temperatures have also been shown to increase the risk of adverse cardiac events and death rates during the winter season in other developed countries [12–14].

Furthermore, cold temperatures may also affect mortality through increased incidence of viral infections, which tend to have a greater impact on vulnerable populations, such as older people [15]. In the first 8 months of 2015, the evidence regarding suspected causes of excess mortality with an inter-seasonal extension [16], divided Italian epidemiologists into two groups. One group argued that the excess winter deaths were linked to the influenza epidemic while summer deaths was caused by the persistent heat wave [16]. The other group attributed the main effect to a specific cohort effect [17].

Limited information is available on the combined effect of influenza, cold weather, and air pollution on mortality, but there is some evidence that air pollution and the weather may affect mortality rates associated with viral epidemics [18]. However, the effect of these 3 exposures on mortality is rarely investigated simultaneously as they are often defined as each other’s confounders [9, 19–21].

In the winter of 2016–2017, there was a significant increase of the number of deaths in Europe [22]; the spike was principally attributed to the influenza epidemic, which occurred 3 weeks earlier than in the previous year [23, 24]. In the province of Milan, a previous, propensity-based report comparing outcomes in vaccinated vs. unvaccinated groups has shown that influenza vaccine is effective at reducing influenza-related mortality by 34% and preventing hospitalizations from complications of influenza by 9% [25]. Therefore, in order to prevent avoidable deaths, evidence on the individual and combined effects of influenza, cold temperatures, and air pollution on mortality would enable public health authorities to promote interventions such increasing influenza vaccination coverage among elderly persons (less than 50% in the province of Milan [26, 27]) and implementing measures to mitigate the effects of environmental risk factors.

The aim of this study was to quantify the excess mortality in winter 2016–2017 in the metropolitan area of Milan and to evaluate the combined short-term effects of low temperatures, influenza, and air pollution.

## Methods

### Setting and participant selection

The area under study is the largest metropolitan population of northern Italy (3,450,000 inhabitants), and includes 195 municipalities covered by the Agency for Health Protection (ATS) of Milan.

All deaths from natural causes among residents aged over 65 years, which occurred between 1 December 2016 and 15 February 2017, were identified. Death information was retrieved from the regional health registry, and the underlying cause of death was collected from the local Register of Death Causes of the ATS. The cause of death was classified according to the ICD-10 codes [28] as being from natural (A00-R99), cardiovascular (I00-I99), or respiratory causes (J00-J99). These causes of death are indeed the most correlated and studied with the exposures considered [7, 9, 10, 20, 25]. The coordinates of the deceased’s residential address were obtained from the regional health registry. Residents with missing address information were assigned as living within the center of the municipality. Neither family members nor the general public were involved in the design or conduct of this study. Ethics approval was not required because evaluation of residents’ use of health services is a statutory function of ATS (L.R. 23/2015), and is a critical component of surveillance and monitoring of the National Healthcare System (NHS). Individuals’ identities were masked by anonymization according to the standard ISO 25237:201. Their unique identification number (fiscal code) was transcoded into a string by the ATS information system, which had no role in analysing the data.

### Exposure variables

Daily environmental data on minimum temperatures and air pollution levels were obtained from the Regional Environmental Protection Agency (ARPA) [29]. As indicator of air pollution, we used the concentration of particulate matter with aerodynamic diameter lower than 10 μm (PM10). In the area covered by ATS, the ARPA has 18 temperature-monitoring stations of which 6 belong to the municipality of Milan. In addition, the ARPA has 24 air pollution-monitoring stations, of which 17 belong to the ATS, and 3 to the municipality of Milan. The coordinates of each monitoring station were obtained from the ARPA website [29, 30]. Each resident who died between 1 December 2016 and 15 February 2017 was associated with the temperature and air pollution reading obtained from the monitoring station at the shortest Euclidean distance from their place of residence. Weekly data on influenza notifications in the region were obtained from the National Health Service Sentinel System (InfluNet) [31, 32]. Epidemiological surveillance in Italy is based on weekly data collected from a sample of general practitioners equally distributed across the country. Incidence rates were expressed as the number of cases per 1000 inhabitants per week. Missing values on a specific day were imputed with the average of all measurements of that exposure for that day collected from the other monitoring stations, weighted by the ratio of the yearly average of that monitoring station over the yearly average of the other monitoring stations, for the same environmental exposure [21, 33].

#### Statistical methods

In order to evaluate the associations between PM10, temperature, and influenza epidemic on mortality, we used a case-crossover, time-stratified design. The study period was divided into monthly strata, and the control days for each case were chosen on the same day of the week in the stratum [20, 34]. Adjustment for usual confounders such as age and gender was ensured by the design. For each exposure, we estimated the odds ratios (OR) with 95% confidence intervals (CI) using conditional logistic regression [35].

As several previous studies of the dose-response relationship between particulate air pollution and mortality [36, 37], had shown a near-linear relationship with no evidence of a risk increase threshold, we assessed exposures as continuous variables in order to determine the shape of the dose-response curve. In addition, we estimated their effect on mortality using dummy variables to evaluate the impact of extreme levels of air pollution, ambient temperature and influenza incidence rates. For this reason, for each outcome we modeled 8 different univariate logistic regression analyses to select the best lag structure for each exposure variable. The first 3 models included each exposure as a continuous variable while 5 models were used to estimate the effect of the exposures on mortality using dummy variables. Following the distributions of variables recorded between 1 December 2016 and 15 February 2017, minimum temperature and influenza rates were dichotomized using median values as cut-off point. For minimum temperature, it was 0 °C. For influenza rates, it was 5 new cases per 10^3^ person/week (Table 1). Concurrently, PM10 was transformed into a dummy variable using the European legal limit of 50, 60, or 70 μg/m^3^. The final lag structure was chosen according to the strength of associations and the width of the confidence intervals [20, 21]. In presence of 2 possible lag structures for the same continuous or dummy exposure, the selection procedure was to choose the concordant lag for the corresponding dummy and continuous exposure. As a result, it should be noted, the first step of the analysis was not to measure the association between exposures and mortality; in addition, each independent regression model only included a single exposure.

**Table 1:** Descriptive statistics of PM10, minimum temperature and influenza rate

					Distance^a^ (Km)	
Exposures	Missing value (%)	Mean ± SD	Min,Max	Median	Mean	Min,Max
Min. Temperature (°C)	4.4	0.22 ± 2.8	−5.5, 6.3	0.03	4.5	0.05, 20.18
PM10 (μg/m^3^)	13	58 ± 26	12.6, 156	55	5	0.03, 16.50
Influenza rates per 10^3^	0	4.9 ± 1.9	1.5, 8.8	5.4		

^a^Distance (Km) between residential address and closest monitoring station

In order to estimate an unbiased association between exposures and mortality, we fitted conditional multivariate logistic regression model, including all exposure variables according to the lag structure identified in the first step. Interactions between exposures were also included. In the multivariate model, to adjust for holidays, we inserted a dummy variable “1” for Italian holidays (6 in the study period) and for the preceding and following days, and “0” for other days. For example, for the Epiphany festivity, a value of “1” was associated to 5th, 6th and 7th of January. The analyses were performed using SAS Software version 9.4 (SAS Institute Inc., Cary, NC, USA).

## Results

### Descriptive statistics

Between 1 December 2016 and 15 February 2017, 7590 residents in the area of the ATS of Milan over 65 years of age died from natural causes. There were 965 (13%) deaths associated with respiratory causes, and 2688 (35%) with cardiovascular causes. Only 14 cases were geo-localized as living within the center of the municipality. The measures of exposure are summarized in Table 1. Of the exposure variables, 4.4% temperature values and 13% of PM10 values were missing, and had to be imputed. The mean distance between the individuals’ place of residence and the nearest temperature or PM10 monitoring station was 4.5 km and 5 km, respectively. Pearson correlation coefficients between pairs of monitoring stations were high for both temperature and PM10, ranging from 0.65 to 0.73, respectively.

### Excess mortality and exposure levels

In comparison to the same period in 2015–2016, excess mortality, among people aged over 65 years, was recorded during the winter of 2016–2017. This excess correspond to 1329 natural deaths, including 381 deaths from respiratory conditions, and 478 deaths from cardiovascular conditions.

**Fig. 1:**
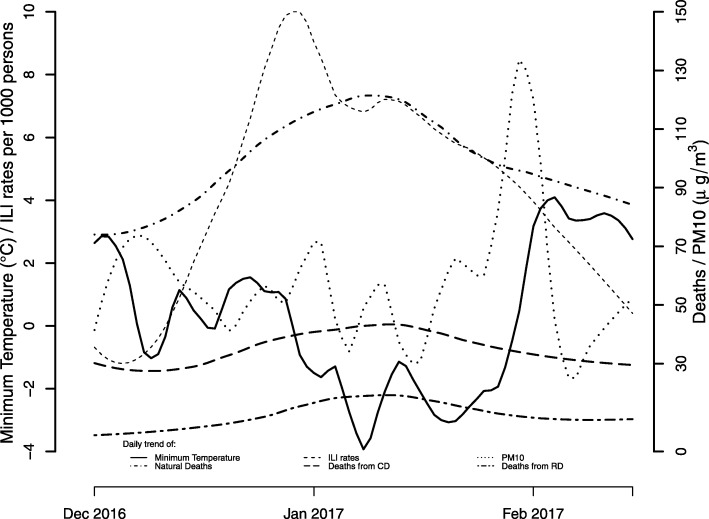
Daily trend of deaths from natural causes, cardiovascular diseases (CD) and respiratory diseases (RD), average concentration of PM10 (μg/m^3^), minimum temperature (°C) and incidence rates of the influenza syndrome (number of new cases every 10^3^ persons)

The standardized mortality ratio (SMR) of dying of natural causes in the period 01/12/2016–15/02/2017 compared to dying of natural causes in the period 01/12/2015–15/02/2016 was 1.21 (95% CI: 1.18–1.23), 1.65 (95% CI: 1.55–1.76) of dying of respiratory causes, and 1.21 (95% CI: 1.17–1.26) of dying of cardiovascular causes. The distribution of influenza rates increased rapidly with a peak at the end of December 2016, natural and cause-specific mortality showed a similar pattern with a peak in January 2017, approximately 2 weeks after the peak in influenza-incidence rates (Fig. 1). During this period, the level of air pollution remained stable at a very high level, with more than 50% of the values> 50 μg/m^3^ and more than 50% of the temperature distribution above 0 °C.

### Lag structure determination

The outputs and the decisions’ rules of the 8 univariate logistic regression models used to determine the lag periods are provided in Supplementary File 1. According to the strength of associations and the width of the confidence intervals, we choose, for the effect of temperature on mortality, a lag period of 6 days for deaths due to natural causes and cardiovascular conditions, and a lag period of 7 days for deaths due to respiratory conditions. For the effect of air pollution on mortality, we selected a lag of 7 days in PM10 for deaths due to natural causes, and cause-specific mortality. Finally, the analyses on influenza have shown a major effect at lag1 for all mortality causes.

### Multivariate models

For continuous exposures (model M1 for natural mortality), we observed an increase of 0.2% in natural mortality associated with a 1 μg/m^3^ increase of PM10 (OR = 1.002; 95% CI: 0.999–1.004) (Table 2). We further observed an increase of 20% (OR = 1.198; 95% CI: 1.156–1.241) associated with 1 case per 10^3^ persons increase in influenza rates, and a decrease of 3.4% (OR = 0.966; 95% CI: 0.944–0.989) associated with 1 °C increase in minimum temperature. For categorical exposures, we observed a statistically significant effect of PM10 on natural mortality (models M3-M4) both for 60 and for 70 μg/m^3^ cut-offs (OR = 1.248; 95% CI: 1.148–1.357 and OR = 1.456; 95% CI: 1.334–1.589, respectively). Influenza and minimum temperature included in the model as dummy variables were significantly associated with natural mortality (models M2-M4). We have found a statistically significant and protective interaction between influenza and minimum temperature (models M2 and M3) on natural mortality.

**Table 2:** Associations between deaths from natural and cause-specific disease and exposures (continuous and dummy)

	Models^a^	PM10	Influenza rates	Temperature	PM10^a^Influenza rates	PM10^a^Temperature	Influenza rates^a^Temperature
NATURAL	M1	1.002 (0.999–1.004)	1.198 (1.156–1.241)	0.966 (0.944–0.989)	1.000 (1.000–1.000)	1.000 (1.000–1.001)	1.000 (0.996–1.004)
	M2	0.956 (0.880–1.038)	1.712 (1.535–1.909)	1.182 (1.051–1.330)	1.058 (0.944–1.186)	1.070 (0.957–1.195)	0.831 (0.732–0.944)
	M3	1.248 (1.148–1.357)	1.827 (1.654–2.019)	1.203 (1.075–1.347)	0.909 (0.810–1.019)	0.948 (0.849–1.059)	0.864 (0.760–0.981)
	M4	1.456 (1.334–1.589)	1.931 (1.753–2.128)	1.164 (1.043–1.299)	0.773 (0.685–0.873)	0.953 (0.846–1.073)	0.881 (0.775–1.002)
CARDIOVASCULAR	M1	0.998 (0.993–1.002)	1.153 (1.088–1.223)	0.961 (0.925–0.999)	1.001 (1.000–1.001)	1.000 (1.000–1.001)	1.003 (0.995–1.010)
	M2	0.860 (0.750–0.990)	1.600 (1.330–1.920)	1.270 (1.040–1.540)	1.220 (1.010–1.480)	0.970 (0.810–1.170)	0.820 (0.660–1.020)
	M3	1.090 (0.950–1.260)	1.680 (1.420–1.990)	1.240 (1.030–1.490)	1.120 (0.920–1.360)	0.910 (0.760–1.100)	0.850 (0.690–1.060)
	M4	1.290 (1.110–1.500)	1.820 (1.540–2.140)	1.170 (0.970–1.400)	0.910 (0.740–1.120)	0.970 (0.790–1.180)	0.880 (0.710–1.090)
RESPIRATORY	M5	1.003 (0.995–1.012)	1.303 (1.166–1.456)	0.931 (0.868–1.000)	0.999 (0.998–1.001)	1.000 (0.999–1.001)	1.006 (0.994–1.018)
	M6	1.080 (0.830–1.390)	2.620 (1.930–3.560)	1.240 (0.830–1.830)	0.730 (0.520–1.040)	1.290 (0.930–1.790)	0.740 (0.500–1.100)
	M7	1.490 (1.150–1.930)	2.680 (2.010–3.580)	1.410 (0.970–2.050)	0.640 (0.450–0.910)	0.950 (0.680–1.320)	0.760 (0.510–1.130)
	M8	1.750 (1.340–2.280)	2.800 (2.110–3.710)	1.380 (0.970–1.970)	0.540 (0.380–0.770)	0.940 (0.660–1.320)	0.760 (0.510–1.130)

Note: All estimates, Odds Ratio (OR) and corresponding 95% confidence intervals, are from multivariate conditional logistic model adjusted for holidays

^a^ M1 contains minimum temperature in continuous (lag6, unit of measurement 1 °C), Influenza rates in continuous (lag1, unit of measurement 1 case per 10^3^ persons), PM10 in continuous (lag7, unit of measurement 1 μg/m^3^) and relative interactions; M2 contains minimum temperature as dummy variable (lag6, cutoff 0 °C), Influenza rates as dummy variable (lag1, 5 case per 10^3^ persons), PM10 as dummy variable (lag7, cutoff 50 μg/m^3^) and relative interactions; M3 contains minimum temperature as dummy variable (lag6, cutoff 0 °C), Influenza rates as dummy variable (lag1, 5 case per 10^3^ persons), PM10 as dummy variable (lag7, cutoff 60 μg/m^3^) and relative interactions; M4 contains minimum temperature as dummy variable (lag6, cutoff 0 °C), Influenza rates as dummy variable (lag1, 5 case per 10^3^ persons), PM10 as dummy variable (lag7, cutoff 70 μg/m^3^) and relative interactions; M5 contains minimum temperature in continuous (lag7, unit of measurement 1 °C), Influenza rates in continuous (lag1, unit of measurement 1 case per 10^3^ persons), PM10 in continuous (lag7, unit of measurement 1 μg/m^3^) and relative interactions; M6 contains minimum temperature as dummy variable (lag7, cutoff 0 °C), Influenza rates as dummy variable (lag1, 5 case per 10^3^ persons), PM10 as dummy variable (lag7, cutoff 50 μg/m^3^) and relative interactions; M7 contains minimum temperature as dummy variable (lag7, cutoff 0 °C), Influenza rates as dummy variable (lag1, 5 case per 10^3^ persons), PM10 as dummy variable (lag7, cutoff 60 μg/m^3^) and relative interactions; M8 contains minimum temperature as dummy variable (lag7, cutoff 0 °C), Influenza rates as dummy variable (lag1, 5 case per 10^3^ persons), PM10 as dummy variable (lag7, cutoff 70 μg/m^3^) and relative interactions

For cardiovascular mortality, we have found an increase of 15.3% (OR = 1.153, 95% CI: 1.088–1.223) associated with an increase of 1 influenza case per 10^3^ persons, and a decrease of 3.9% (OR = 0.961, 95% CI: 0.925–0.999) associated with 1 °C increase in minimum temperature (model M1 on cardiovascular mortality). A significant direct association between PM10 and mortality of cardiovascular diseases (model M4) was found for PM10 with 70 μg/m^3^ as cut-off (OR = 1.290, 95% CI: 1.110–1.500). The effects of influenza and minimum temperature (dummy variables) on mortality for cardiovascular diseases (models M2-M4) were statistically significant in all tested cases. We found a statistically significant and positive interaction effect of PM10 (50 μg/m^3^ cut-off) and influenza on mortality for cardiovascular diseases (model M2).

On respiratory mortality (model M5), there was an overall increase of 0.3% (OR = 1.003, 95% CI: 0.995–1.012) associated with a 1 μg/m^3^ increase of PM10. Meanwhile, the risk of respiratory mortality increased by 30% for 1 influenza case per 10^3^ persons (OR = 1.303, 95% CI: 1.166–1.456). An association between PM10 dichotomized as 60 and 70 μg/m^3^ for respiratory mortality (models M7-M8) was found (OR = 1.490, 95% CI: 1.150–1.930 and OR = 1.750, 95% CI: 1.340–2.280, respectively). Influenza was always significantly associated with mortality for respiratory causes (models M6-M8).

## Discussion

The 2016–2017 winter was characterized by a significant increase in total mortality, especially among people aged over 65 years, although some variation was reported between European countries. This spike in mortality was principally attributed to influenza and cold temperatures. In Italy, the mortality peak was similar and concomitant with that observed in many European countries such as Spain and France [22].

In our study, influenza and temperature were consistently associated with deaths from natural causes, cardiovascular conditions, and respiratory conditions. Air pollution, measured as continuous variable, was associated with increased risk of death due to natural causes and respiratory mortality.

In addition, our results suggest that an increase in deaths due to natural causes and respiratory mortality are associated with high levels of PM10 (60 and 70 μg/m^3^). PM10 was associated with mortality for cardiovascular diseases in the model where PM10 was dichotomized for levels greater than 70 μg/m^3^. This high reference threshold is probably attributable to the high mean concentration (> 50 μg/m^3^) over the study period. These results indicate that very high levels of air pollution have adverse health effects, and these adverse effects are aggravated by cold weather, a situation that is becoming common in winter in many metropolitan areas. A major strength of our study was that it assessed the combined effect of air pollution, cold weather, and influenza on deaths from natural causes and cause-specific mortality.

Previous studies have found that an increase of 10 μg/m^3^ in PM10 was associated with 0.3 to 0.9% increase in deaths due to natural causes [7, 21, 33, 38] and that a decrease in ambient temperature of 1 °C was associated with 0.7 to 1.35% increase in deaths due to natural causes [9, 39].

Regarding mortality due to cardiovascular conditions, previous studies have shown that an increase of 10 μg/m^3^ in PM10 was associated with a 0.54 to 0.93% increase in cardiovascular deaths [7, 33]. There have been several studies of the association between cardiovascular mortality and cold temperatures: a decrease of 1 °C has been reported to be associated with an increase of 1.72% in cardiovascular mortality [9], an increase of 0.7–0.8% with coronary events [12] and, for total myocardial infarction, a 10 °C decrease in 5-day average temperature was associated with a relative risk of 1.10 [13].

An increase of 10 μg/m^3^ in PM10 has been reported to be associated with 0.54 to 2.29% increase in mortality due to respiratory conditions [7, 21, 33]. However, the results vary among studies and are not directly comparable because of differences in the number of lag days across studies.

Influenza epidemics remain a major cause of morbidity and mortality during the winter season [16, 40]. However, to our knowledge, no previous studies have focused directly on the association between influenza incidence rates and mortality, as most previous studies of influenza and mortality have focused on the air pollution-mortality relationship and have treated influenza as a confounder.

Knowledge of the combined effect of air pollution, low temperature and influenza on mortality is limited. Analitis et al. [18] studied the interaction between air pollution and apparent temperature on mortality in nine European cities between 2004 and 2010. They found a significant and positive interaction between high level of PM10 and apparent temperature on mortality for all ages combined, and for those aged ≥75 years.

In addition, extreme temperatures are related to global warming and other phenomena, including air pollution. Evidence that health hazards associated with air pollution, influenza, and low temperature might increase as a result of interaction between the three exposures may open new research avenues within the fields of climate change, air pollution, infectious disease, and public health. Global climate change has led to phenomena such as El Niño Sothern Oscillations (ENSO), which may affect the climate in many parts of the world, including Europe between November and December [41]. A connection between strong cold ENSO phases and lower temperature in European winters has been highlighted [19, 42]. In East Asia [43–45] and America [42] the ENSO has also been associated with precipitation, temperature variation [46], and pollution. Climate affects the range of infectious diseases likely to occur in a particular region, whereas the weather affects the timing and intensity of specific disease outbreaks [47]. Several authors have suggested the role of ENSO in infectious diseases [48], but also pandemic [49] and epidemic influenza [50]. Therefore, it would be interesting to estimate the impact of climate change on the mortality excess occurred in the winter of 2016–2017.

### Strengths and limitations

One of the major strength of this study is the size of the population investigated as the total number of deaths among people over 65 years of age was considered, without any selection criteria. Furthermore, the case-crossover study design assures adjustment for important confounders that are known to bias the effect of air pollution, cold temperature and influenza on mortality such as gender, age and most importantly individual socio-economic deprivation.

However, this study has several limitations. Firstly, the use the ARPA monitoring stations remote from the place of residence for data regarding PM10 and temperature, may not have provided an accurate estimate of these exposures in the direct proximity. The variability between the place of residence and the nearest monitoring station ranged from 0.05–20.18 km for temperature monitoring stations, and 0.03–21.78 km for PM10 monitoring stations. Further studies that use more precise measurements of environmental factors, covering a greater geographic area, are required. These could include pollutant maps produced with satellite data to estimate level of air pollution [51]. Most studies of adverse health effects attributable to air pollution have used PM10 as the indicator of air pollution. It would be worthwhile to evaluate the effect of other air pollution indicators, and their effect in combination with temperature and influenza, on mortality using multipollutant models. Secondly, although our findings are consistent with those found in previous studies, they may be partially distorted due to the number of tests performed. Our study focused specifically on the winter of 2016–2017 and did not include any comparisons with previous years. Incorporating data from other winter period in the analysis could make the results more generalizable.

## Conclusions

This study confirmed influenza as contributed to the excess mortality that occurred in the winter of 2016–2017 in the metropolitan area of Milan. Vaccination coverage of elderly persons should continue as to be the primary strategy for preventing influenza-associated deaths. However, our results show that the excess mortality during the winter of 2016–2017 was also related to air pollution and cold temperatures. Here we suggested a combined effect on natural, cardiovascular and respiratory mortality, which highlight the need of specific policies not only intended to increment the vaccination coverage but also on mitigating the effects of environmental risk factors in metropolitan areas.

## Supplementary information

**Additional file 1.** Associations between deaths from natural and cause-specific disease and exposures (continuous and dummy) at various lags.

## Data Availability

The environmental dataset analysed during the current study are available from the ARPA (www.arpalombardia.it) and InfluNet (https://www.epicentro.iss.it/influenza/influnet) websites. Daily aggregated mortality data can be available upon request to the corresponding author.

## Abbreviations

ARPA: Regional Environmental Protection Agency
ATS: Agency for Health Protection of Milan
CD: Cardiocirculatory diseases
InfluNet: National Health Service Sentinel System
RD: Respiratory diseases
