# Supplementary material for: Effects of pollution, low temperature and influenza syndrome on the excess mortality risk in winter 2016–2017
Source: BMC Public Health. 2019 Nov 4;19:1445. doi: 10.1186/s12889-019-7788-8 (PMC6829994; doi:10.1186/s12889-019-7788-8)
Supplement: Supplementary file 1 — Additional file 1. Associations between deaths from natural and cause-specific disease and exposures (continuous and dummy) at various lags. [file 12889_2019_7788_MOESM1_ESM.docx]

# ADDITIONAL FILES

|  | OR (95% CI) at various lag | | | | | | | | | | | |
| --- | --- | --- | --- | --- | --- | --- | --- | --- | --- | --- | --- | --- |
|  | 0 | 1 | 2 | 3 | 4 | 5 | 6 | 7 | 0-1 | 0-2 | 3-5 | 1-6 |
| NATURAL |  |  |  |  |  |  |  |  |  |  |  |  |
| Min. Temp*^a^* | 1.01 (1.00-1.02) | 1.01 (1.00-1.02) | 1.01 (1.00-1.02) | 0.99 (0.98-1.00) | 0.98 (0.97-0.99) | 0.98 (0.97-0.99) | 0.98 (0.97-0.99) | 0.98 (0.97-0.99) |  | 1.02 (1.00-1.03) | 0.97 (0.96-0.99) | 0.97 (0.95-0.99) |
| Influenza rates*^b^* | 1.12 (1.10-1.13) | 1.18 (1.16-1.20) |  |  |  |  |  |  | 1.16 (1.14-1.18) |  |  |  |
| PM10*^c^* | 0.997 (0.996-0.998) | 0.999 (0.998-1.000) | 1.000 (0.999-1.001) | 1.001 (1.001-1.002) | 1.002 (1.001-1.003) | 1.003 (1.002-1.004) | 1.003 (1.002-1.004) | 1.004 (1.003-1.004) |  | 0.998 (0.997-0.999) | 1.003 (1.002-1.004) | 1.003 (1.001-1.004) |
| Min. Temp*^d^* | 1.03 (0.96-1.11) | 0.99 (0.92-1.07) | 1.00 (0.93-1.07) | 1.10 (1.02-1.18) | 1.10 (1.03-1.18) | 1.12 (1.05-1.19) | 1.17 (1.10-1.25) | 1.14 (1.07-1.21) |  | 1.05 (0.97-1.13) | 1.11 (1.03-1.20) | 1.11 (1.03-1.21) |
| Influenza rates*^e^* | 1.32 (1.24-1.42) | 1.67 (1.56-1.80) |  |  |  |  |  |  | 1.28 (1.18-1.38) |  |  |  |
| PM10 ≥50*^f^* | 0.74 (0.70-0.78) | 0.80 (0.76-0.84) | 0.84 (0.79-0.88) | 0.85 (0.81-0.89) | 0.89 (0.84-0.93) | 0.95 (0.90-0.99) | 0.98 (0.94-1.03) | 1.06 (1.01-1.12) |  | 0.78 (0.74-0.82) | 0.86 (0.82-0.90) | 0.86 (0.82-0.91) |
| PM10 ≥60*^g^* | 0.77 (0.73-0.81) | 0.85 (0.81-0.90) | 0.88 (0.83-0.92) | 0.92 (0.88-0.97) | 0.96 (0.92-1.01) | 1.02 (0.97-1.08) | 1.08 (1.03-1.13) | 1.16 (1.11-1.22) |  | 0.80 (0.76-0.85) | 0.94 (0.89-0.99) | 0.92 (0.87-0.96) |
| PM10 ≥70*^h^* | 0.79 (0.75-0.83) | 0.91 (0.86-0.96) | 0.91 (0.87-0.96) | 0.99 (0.94-1.05) | 1.03 (0.97-1.08) | 1.12 (1.06-1.18) | 1.16 (1.10-1.22) | 1.22 (1.16-1.29) |  | 0.85 (0.80-0.90) | 1.05 (0.99-1.11) | 1.01 (0.96-1.07) |
| CARDIOVASCULAR |  |  |  |  |  |  |  |  |  |  |  |  |
| Min. Temp*^a^* | 1.01 (1.00-1.03) | 1.01 (0.99-1.03) | 1.01 (0.99-1.03) | 0.99 (0.97-1.00) | 0.98 (0.96-1.00) | 0.98 (0.97-1.00) | 0.99 (0.97-1.00) | 0.99 (0.97-1.00) |  | 1.02 (1.00-1.05) | 0.97 (0.95-1.00) | 0.98 (0.95-1.01) |
| Influenza rates*^b^* | 1.11 (1.08-1.14) | 1.18 (1.15-1.21) |  |  |  |  |  |  | 1.15 (1.12-1.19) |  |  |  |
| PM10*^c^* | 0.996 (0.995-0.998) | 0.999 (0.997-1.000) | 0.999 (0.998-1.001) | 1.001 (0.999-1.002) | 1.001 (0.999-1.002) | 1.002 (1.000-1.003) | 1.002 (1.000-1.003) | 1.002 (1.001-1.004) |  | 0.998 (0.996-0.999) | 1.002 (1.000-1.003) | 1.001 (0.999-1.003) |
| Min. Temp*^d^* | 1.01 (0.90-1.14) | 1.00 (0.89-1.13) | 1.00 (0.89-1.13) | 1.15 (1.02-1.29) | 1.05 (0.94-1.17) | 1.10 (0.99-1.23) | 1.17 (1.06-1.30) | 1.10 (0.99-1.22) |  | 1.07 (0.94-1.22) | 1.13 (1.00-1.27) | 1.07 (0.93-1.23) |
| Influenza rates*^e^* | 1.37 (1.22-1.53) | 1.70 (1.51-1.91) |  |  |  |  |  |  | 1.34 (1.17-1.53) |  |  |  |
| PM10 ≥50*^f^* | 0.76 (0.69-0.83) | 0.79 (0.73-0.87) | 0.84 (0.77-0.92) | 0.82 (0.75-0.89) | 0.79 (0.72-0.86) | 0.92 (0.84-0.99) | 0.90 (0.83-0.98) | 0.99 (0.91-1.08) |  | 0.77 (0.71-0.84) | 0.80 (0.73-0.87) | 0.78 (0.71-0.85) |
| PM10 ≥60*^g^* | 0.76 (0.70-0.83) | 0.83 (0.76-0.91) | 0.85 (0.78-0.93) | 0.90 (0.83-0.98) | 0.88 (0.81-0.96) | 0.99 (0.91-1.08) | 1.01 (0.93-1.10) | 1.13 (1.04-1.22) |  | 0.81 (0.75-0.89) | 0.88 (0.81-0.95) | 0.85 (0.78-0.93) |
| PM10 ≥70*^h^* | 0.78 (0.71-0.86) | 0.91 (0.83-1.00) | 0.89 (0.82-0.98) | 0.97 (0.88-1.06) | 0.98 (0.90-1.08) | 1.05 (0.95-1.15) | 1.07 (0.98-1.18) | 1.19 (1.09-1.31) |  | 0.84 (0.76-0.93) | 1.00 (0.91-1.10) | 0.96 (0.88-1.06) |
| RESPIRATORY |  |  |  |  |  |  |  |  |  |  |  |  |
| Min. Temp*^a^* | 1.01 (0.97-1.04) | 1.00 (0.97-1.04) | 0.99 (0.96-1.03) | 0.99 (0.95-1.02) | 0.98 (0.95-1.02) | 0.98 (0.95-1.01) | 0.97 (0.95-1.00) | 0.96 (0.93-0.98) |  | 1.00 (0.96-1.05) | 0.97 (0.93-1.01) | 0.95 (0.90-1.01) |
| Influenza rates*^b^* | 1.19 (1.14-1.24) | 1.25 (1.19-1.31) |  |  |  |  |  |  | 1.25 (1.19-1.31) |  |  |  |
| PM10*^c^* | 0.996 (0.994-0.998) | 0.999 (0.997-1.001) | 1.001 (0.999-1.004) | 1.002 (0.999-1.004) | 1.001 (0.999-1.003) | 1.001 (0.999-1.004) | 1.002 (0.999-1.004) | 1.004 (1.001-1.006) |  | 0.998 (0.995-1.001) | 1.002 (0.999-1.005) | 1.002 (0.999-1.005) |
| Min. Temp*^d^* | 1.07 (0.88-1.30) | 1.07 (0.87-1.31) | 1.02 (0.83-1.25) | 1.03 (0.84-1.25) | 1.04 (0.86-1.26) | 1.18 (0.99-1.42) | 1.18 (0.99-1.40) | 1.32 (1.11-1.57) |  | 1.16 (0.93-1.45) | 1.17 (0.95-1.44) | 1.14 (0.91-1.44) |
| Influenza rates*^e^* | 1.50 (1.21-1.84) | 2.17 (1.77-2.68) |  |  |  |  |  |  | 1.61 (1.28-2.03) |  |  |  |
| PM10 ≥50*^f^* | 0.68 (0.59-0.79) | 0.85 (0.73-0.98) | 0.87 (0.75-1.00) | 0.82 (0.72-0.95) | 0.86 (0.75-0.99) | 0.87 (0.75-0.99) | 0.96 (0.84-1.10) | 1.08 (0.94-1.24) |  | 0.82 (0.71-0.95) | 0.80 (0.70-0.93) | 0.85 (0.74-0.99) |
| PM10 ≥60*^g^* | 0.73 (0.63-0.85) | 0.93 (0.80-1.07) | 0.94 (0.82-1.09) | 0.90 (0.78-1.04) | 0.98 (0.86-1.13) | 0.96 (0.84-1.10) | 1.04 (0.91-1.20) | 1.13 (0.98-1.29) |  | 0.83 (0.72-0.96) | 0.87 (0.76-1.00) | 0.85 (0.74-0.98) |
| PM10 ≥70*^h^* | 0.80 (0.68-0.93) | 0.93 (0.80-1.08) | 1.04 (0.89-1.21) | 0.97 (0.83-1.13) | 1.01 (0.86-1.17) | 1.07 (0.92-1.24) | 1.18 (1.01-1.38) | 1.18 (1.01-1.37) |  | 0.87 (0.74-1.02) | 1.01 (0.86-1.18) | 1.01 (0.86-1.18) |

**Additional file 1**. Associations between deaths from natural and cause-specific disease and exposures (continuous and dummy) at various lags.

Note: All estimates, Odds Ratio (OR) and corresponding 95% confidence intervals, are from univariate conditional logistic model.

*^a^*minimum temperature in continuous (1°C).

*^b^*Influenza rates in continuous (1 case per 10^3^ persons).

*^c^*PM10 in continuous (1 μg/m^3^).

*^d^*minimum temperature as dummy variable (0°C).

*^e^*Influenza rates as dummy variable (5 case per 10^3^ persons).

*^f^*PM10 as dummy variable (50 μg/m^3^).

*^g^*PM10 as dummy variable (60 μg/m^3^).

*^h^*PM10 as dummy variable (60 μg/m^3^).

***Lag structure determination***

*Temperature*

Death rates attributable to natural causes, respiratory conditions, and cardiovascular conditions decreased with an increase in the minimum temperature when the minimum temperature was considered as a continuous variable. In contrast, when the temperature variable was dichotomized, the analysis of the lag structure (expressed in days) revealed a prolonged effect of temperature on both natural deaths and cardiovascular mortality from lag3 up to lag7, and for cumulative lags 3-5, 1-6. The lag structure suggested a lag period of 7 days for the effect of temperature on respiratory mortality. Therefore, in order to model the effect of temperature on mortality, we selected a lag period of 6 days for deaths due to natural causes and cardiovascular conditions, and a lag period of 7 days for deaths due to respiratory conditions.

*Air pollution*

The analysis of PM10 as a continuous variable showed a prolonged effect of air pollution on natural mortality starting at 3 days lag up to 7 days lag and for cumulative lags of 3–5 days, and 1–6 days. The results were similar for the lags between air pollution, cardiovascular and respiratory mortality. The strongest effect of dichotomized PM10s was observed at 7 days for deaths due to natural causes and cause-specific mortality. Therefore, we selected a lag of 7 days in PM10 for deaths due to natural causes, and cause-specific mortality.

*Influenza*

The analyses on influenza have shown a major effect at lag1 for all mortality causes.
